# Visualizing Reach of Racial and Ethnic Approaches to Community Health for Asian Americans: the REACH FAR Project in New York and New Jersey

**DOI:** 10.5888/pcd15.180026

**Published:** 2018-09-13

**Authors:** Susan S. Kum, Shilpa Patel, Mary Joy Garcia, Sara S. Kim, Rucha Kavathe, Catherine Choy, MD Taher, Stella S. Yi, Nadia Islam, Simona C. Kwon

**Affiliations:** 1 Department of Population Health, NYU School of Medicine, New York, New York; 2 Kalusugan Coalition, Inc., Woodside, New York; 3 Korean Community Services of Metropolitan NY, Inc., New York, New York; 4 UNITED SIKHS, New York, New York

**Figure:**
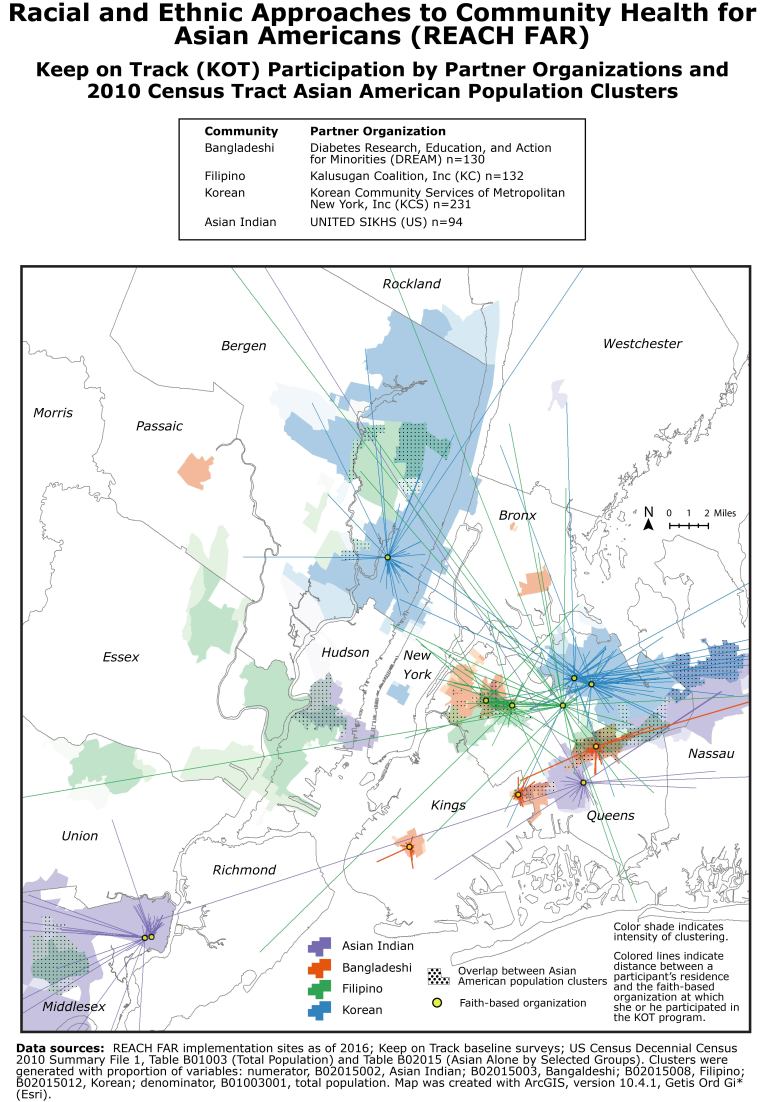
The REACH FAR (Racial and Ethnic Approaches to Community Health for Asian Americans) project in New York and New Jersey is guided by a multisector coalition made up of the New York University Center for the Study of Asian American Health, the New York City Department of Health and Mental Hygiene, 4 community-based organizations: UNITED SIKHS, serving the Asian Indian population; Diabetes Research, Education, and Action for Minorities, serving the Bangladeshi community; Kalusugan Coalition, Inc., serving the Filipino community; and Korean Community Services of Metropolitan New York, Inc., serving the Korean community; and other organizations and groups serving these communities. The map shows locations in 2016 of faith-based organizations that served as implementation sites for Keep on Track, an intervention consisting of nutrition and hypertension management/cardiovascular disease prevention strategies for Asian American communities. Implementation sites are located in Asian American population clusters. Lines on the map, representing the number of participants in the Keep on Track program, provide a visual representation of service delivery and reach of the REACH FAR project.

## Background

A growing body of literature shows that health promotion and disease prevention strategies and messages may not be effective in reaching racially and ethnically diverse communities, unless those strategies are culturally and linguistically adapted for target communities (1). The Racial and Ethnic Approaches to Community Health for Asian Americans (REACH FAR) project is a multilevel, evidence-based program of health promotion and disease prevention for Asian American communities in New York and New Jersey. Guided by a socio-ecological framework, social marketing principles, and a community based participatory approach, the project implemented multilevel, evidence-based strategies culturally adapted to address hypertension and improve access to healthy food options for Asian Americans in various community settings (1). The strategies were delivered through a multisector coalition made up of a lead academic agency, New York University Center for the Study of Asian American Health; the NYC Department of Health and Mental Hygiene (NYC DOHMH); 4 community-based organizations: UNITED SIKHS, serving the Asian Indian population; Diabetes Research, Education, and Action for Minorities, serving the Bangladeshi community; Kalusugan Coalition, Inc., serving the Filipino community; Korean Community Services of Metropolitan New York, Inc., serving the Korean community; and other organizations and groups serving these communities. Coalition partners implemented strategies at various community sites, including faith-based organizations, ethnic restaurants, grocery stores, pharmacies, and primary care practices.

REACH FAR has 2 program arms to deliver culturally and linguistically adapted resources. The first arm is focused on improving access to healthy foods and beverages and includes implementation of policies adapted for communal meals at faith-based organizations, healthy food options and labeling at restaurants, and strategic placement and discounting of healthy food products at grocery stores (2). The second arm is focused on improving access to hypertension management and cardiovascular disease prevention programs by offering the NYC DOHMH’s Keep on Track (KOT) blood-pressure monitoring program at faith-based organizations and increasing access to Million Hearts (https://millionhearts.hhs.gov/) blood pressure medication adherence resources at faith-based sites, community-based pharmacies, and health care providers’ offices (3). The REACH FAR coalition leveraged existing partnerships and fostered new relationships with implementation sites to tailor and disseminate strategies for their respective communities.

Geographic areas with high concentrations of targeted Asian American communities were selected at project conception. Hence, a primary motivation for creating our map was to assess the population and geographic reach of the REACH FAR project by comparing Asian American population clusters (ie, geographic areas with high concentrations of Asian Indian, Bangladeshi, Filipino, and Korean populations) with the locations of implementation sites and participants. A desire lines (or spider diagram) approach was used to visualize KOT program participation. Mapping provides a visual means of evaluating program reach and is a concise way of communicating the role of implementation sites in delivering culturally, linguistically, and geographically targeted health prevention strategies.

## Methods

We geocoded the addresses of 12 implementation sites active as of 2016 and residential addresses of KOT program participants reported in baseline surveys from 2015 and 2016 (4–6). All research conducted with human participants was reviewed by the NYU School of Medicine Institutional Review Board and approved as an expedited study. Data processing was performed with version R version 3.4.1 (R Foundation for Statistical Computing). Straight lines were created between successfully geocoded participants’ residences (n = 587; 86% of participants) and faith-based organizations (n = 12); each line represents a KOT program participant (7). Population estimates from Census 2010 Summary File 1 data and household language from ACS 2015 5-Year estimates were obtained for Census tracts in NY and NJ (8,9). Processed data were exported as Esri shape files (10). The map construction and geo-processing related to population clusters were completed with ArcGIS 10.4.1 (Environmental Systems Research Institute [Esri]). The Asian American subgroup population clusters are significant hot spots, positive *z* scores, determined by the ArcGIS Hot Spot Analysis (Getis-Ord Gi*) tool (Esri) with the Contiguity Edges Corner and Apply False Discovery Rate correction parameters; the shading indicates intensity of clustering with darker shading indicating more clustering of high values (larger *z* scores). Overlaps in population clusters were found by using the ArcMap Intersect tool, version 10.3 (Esri).

## Main Findings

An overarching objective of REACH FAR is delivery of targeted strategies for 4 Asian American communities. The map demonstrates that program implementation sites are located in population clusters of targeted Asian American subgroup populations, as intended at program inception: Asian Indian in Middlesex and Queens counties, Bangladeshi in Kings and Queens counties, Filipino in Queens county, and Korean in Queens and Bergen counties. By mapping KOT program participants, we found that large concentrations of congregants residing in areas neighboring each faith-based site are reached by the KOT program. However, the maps also demonstrate that the programs also reached community members residing outside of immediate or neighboring ethnic enclaves. We suspect that people attending these events prefer or need culturally and linguistically adapted resources that are unavailable in the neighborhoods in which they reside. Culturally and linguistically adapted materials regarding healthy eating and blood pressure control developed for limited English-proficient Asian Americans were disseminated at implementation sites (11), reaching 1,353,201 people as of September 30, 2017. The map reveals opportunities for collaboration, the areas where population clusters overlap, and gaps in coverage in areas with population clusters where the project did not have implementation sites.

## Action

Our map provides a visual illustration of the network of faith-based organizations and community-based organizations coordinating to promote healthy eating and heart health and the progress of implementation as illustrated with the KOT program. To this end, mapping products are being used in the following ways to enhance future coordination and collaboration between partners:

1. The map is being presented to the NYC DOHMH to demonstrate the need for reaching Asian American populations. Before our coalition efforts, the KOT program had not been implemented in any faith-based organizations serving Asian Americans. Our results demonstrate both success and potential for future NYC DOHMH engagement efforts with Asian American communities.
2. The map is being reviewed at coalition meetings to discuss opportunities for scaling the program to expand program reach. For example, the map suggests that partnering with community-based organizations serving the Asian Indian and Filipino communities in Hudson County or the Bangladeshi community in Bronx County may expand reach.
